# Controversies and clinical unknowns in the use of PARP inhibitors in ovarian cancer

**DOI:** 10.1177/17588359251343973

**Published:** 2025-06-14

**Authors:** Uma A. Mukherjee, Rowan E. Miller, Jonathan A. Ledermann

**Affiliations:** Cancer Research UK and UCL Cancer Trials Centre, UCL Cancer Institute, University College London, London, UK; University College London Hospital, London, UK; University College London Hospital, London, UK; St Bartholomew’s Hospital, London, UK; Department of Oncology, UCL Cancer Institute, University College London, Paul O’Gorman Building, 72 Huntley Street, London WC1E 6DD, UK

**Keywords:** DNA repair mechanism mutations, homologous recombination deficiency (HRD), ovarian cancer, PARP inhibitor, translational research, tumour biology

## Abstract

Poly (adenosine diphosphate-ribose) polymerase inhibitors (PARPi) have significantly improved the treatment of advanced ovarian cancer, however, there are still many aspects of their use that require further understanding. The optimal duration, timing and dosage of these agents and how to manage (oligo) progression occurring both during and following PARPi therapy are discussed. The evidence supporting their rechallenge, and how to overcome resistance are addressed. The long-term impacts of PARPi and monitoring patients during therapy are all important research themes to expand on.

## Plain language summary

PARP inhibitors: challenges and uncertainties within ovarian cancer

PARP inhibitors are tablet medications used to treat ovarian cancer and have significantly improved the survival prospects of women with the disease, especially if they have a BRCA gene mutation. As their use has become more widespread, some challenges and uncertainties have emerged. These include the optimum length of time that these drugs should be used, both when used as treatment to prolong the response after first-line chemotherapy or to treat disease that has later progressed (relapsed) after chemotherapy. The degree of benefit of PARPi in patients without a BRCA mutation remains less clear. It is also not known whether PARPi could be beneficial if used before ovarian cancer surgery, and clinical studies are underway to assess this. While PARPi can be effective initially, many patients will develop resistance to these drugs, leading to relapse of their cancer. Proventing or overcoming this remains a key challenge. Re-use of the PARPi, when the amount of the disease at relapse is small, or re-using PARPi after radiotherapy or surgery, have been explored but the benefit is not yet clear. Similarly, combining the PARPI and another drug to prevent resistance is currently under investigation. The best way to monitor patients on PARPi for cancer relapse is also an area of uncertainty report. The full extent of long-term toxicities is becoming apparent as clinical studies report long-term data. Future research in ovarian cancer should focus on resolving ways to overcome the resistance associated with PARPi, developing more precise ways to select for those patients who would benefit most from their use. Addressing these challenges and unknowns will be important for optimising the use of PARPi in ovarian cancer treatment and further improving survival rates.

## Introduction

Poly (adenosine diphosphate-ribose) polymerase inhibitors (PARPi) have significantly improved the outcome of women with ovarian, fallopian tube or primary peritoneal cancer (subsequently referred to as ovarian cancer, OC) in response to platinum-based chemotherapy (PBC) and have transformed the treatment landscape of this disease, particularly in the first-line maintenance setting.^1–3^ Based on the results of many randomised phase III studies,^4–6^ several PARPi are now licenced as maintenance therapy in both the first-line and relapsed platinum-sensitive OC settings.^7–9^ Despite their clinical benefit, questions remain about the optimum treatment duration, monitoring frequency with blood tests, biomarkers and imaging during treatment, and how to manage patients who progress on or after treatment. Furthermore, disease progression still occurs in many patients and there is a need to develop better treatments. Such strategies might include PARPi rechallenge, or combination therapies to prevent or overcome resistance. These will be discussed further in this review.

### Practicalities of PARPi therapy: When/how long/what dose?

#### When to initiate PARPi

Randomised phase III trials demonstrated a benefit from maintenance therapy with PARPi in patients with platinum-sensitive recurrence (SOLO2/NOVA/ARIEL3).^5,10,11^ In some cases, long-term gains were seen with patients remaining on PARPi with continuing benefit for more than 5 years. More recently, trials have explored PARPi therapy in the front-line setting, with significant improvements in Progression-Free Survival (PFS).^12–14^ This is particularly so for tumours with a Breast Cancer Susceptibility Gene (BRCA) mutation (BRCAm) or with homologous recombination deficiency (HRD) in repair of DNA (HRD positive). Overall survival (OS) results are now appearing although they less easy to interpret as they are secondary endpoints and many patients in the control arms subsequently received PARPi. In patients with a BRCAm receiving olaparib after front-line chemotherapy in the SOLO1 trial, there was a survival benefit, with a sustained improvement at 7 years.^14^ A survival benefit was also seen in the BRCAm exploratory subgroup in PAOLA-1 combining olaparib and bevacizumab.^12^ However, this was not the case in patients with a BRCAm who received niraparib in the PRIMA study.^15^ Similarly, there was an OS benefit in the HRD-positive exploratory subgroups in the PAOLA-1 study, again not seen in the PRIMA trial. The reasons for these differences present in similar biomarker subgroups that had a BRCAm, or with HRD-positive tumours are unclear, suggesting that other factors may have affected the results. These include differences in prognostic characteristics of the patients entered as well as confounding factors such as the percentage of patients in the control arms who crossed over to a PARPi at relapse. PRIMA included patients with worse clinical characteristics and who were therefore at the highest risk of recurrence. In total, 35.1% of patients had stage 4 disease (compared to 15% in SOLO1 and 30% in PAOLA-1). 66.7% of patients received neoadjuvant chemotherapy (% unknown in SOLO1 and PAOLA-1), indicating that primary debulking surgery was not feasible due to the extent of their disease. Patients who had FIGO stage 3 disease needed to have gross residual disease after primary debulking surgery and there were fewer patients who had upfront surgery. In contrast, most (44%) stage 3 patients in SOLO-1 underwent upfront debulking surgery and had no visible residual disease. Although formal crossover between the treatment groups was not permitted within SOLO1 or PRIMA, patients could receive subsequent therapies at the investigators’ discretion following study treatment discontinuation. 44.3% of patients in the placebo group of SOLO1 received a PARPi in a subsequent line of therapy with 24.4% receiving it as the next subsequent therapy.^14^ In PAOLA-1, 55% of *BRCA*m patients and 51% of HRD-positive patients in the placebo arm received PARPi in subsequent lines.^12^ Subsequent therapy with PARPi occurred in 37.8% of patients in the placebo group overall, 57.7% of patients *BRCA*m tumours and 48.8% of patients with HRD-positive tumours. PARPi was given to 46.5% of *BRCA*m patients and 37.3% of HRD-positive patients after their next subsequent therapy. The effectiveness of subsequent PARPi therapy may have diluted the treatment effect observed with niraparib in the placebo arm. However, the similar values across the trials do not completely account for the discrepancy between OS benefits across the three studies. The extended duration of maintenance PARPi therapy in PRIMA compared to SOLO1 (3 vs 2 years) may have lessened the response to subsequent platinum retreatment, possibly due to cross-resistance, as described in SOLO2.^16^ The higher rates of dose interruption in the niraparib group (80.8%), compared to 23% in the placebo group, might have resulted in greater censoring of the niraparib group, potentially skewing the PFS curves in favour of PARPi. This could also impact on full exposure to both the PARPi and subsequent therapies and thereby negatively impacting OS.^17^ It is worth noting, that in the earlier studies with PARPi in recurrent OC, where crossover was less due to a lower availability of PARPi, there were larger differences in OS. For example, in study 19, a randomised phase II trial of olaparib maintenance in platinum-sensitive recurrent ovarian cancer (PSROC), there was a numerical benefit in OS in favour of olaparib and crossover occurred in only 12% of patients.^18^ In contrast, in the ARIEL3 trial with rucaparib in recurrent OC where no benefit in OS was seen, the crossover rate to a PARPi in the control arm was 71%.^19^ Nevertheless, even in the absence of an OS benefit, subgroups with a BRCAm or HRD positivity in PRIMA or ATHENA-mono^20^ (with rucaparib) had a sustained and clinically significant benefit in PFS, which was the primary endpoint of these trials. However, the OS results raise the question of whether it is better to use a PARPi in the front-line setting to prolong first-line remission with later relapse, or after a relapse. It is worth noting that some patients remain free from recurrence without a PARPi. In SOLO2, 27% of participants in the placebo group were free of PFS events at 3 years,^1^ and after a median follow-up of 5 years, the median PFS was 13.8 months in the placebo group.^21^ Notwithstanding these results, most clinicians favour the use of a PARPi in the front-line setting in patients with a BRCAm or HRD positive tumours.

The role of PARPi in tumours that are HRD negative (sometimes called homologous recombination proficient) is less clear. Both the PRIMA and ATHENA-mono trials demonstrated small benefits in PFS in the HRD-negative groups when niraparib or rucaparib, respectively, were used versus placebo.^2,3^ No benefit was seen in this biomarker subgroup receiving the combination of olaparib and bevacizumab in PAOLA-1.^22^ The benefit in PFS is shorter than in HRD-positive tumours, although there is a small cohort of patients with a long duration of response. It remains unclear whether PARPi should be used in this subgroup, which comprises 50% of patients with high-grade tumours, and if so, in whom. One area being explored is whether the response to PARPi in HRD-negative tumours can be improved by adding an immune checkpoint inhibitor (ICI). Results of the DUO-O study, which investigated the combination of chemotherapy, bevacizumab and durvalumab followed by the addition of olaparib as first-line maintenance therapy in patients with newly diagnosed advanced BRCAwt OC, showed an improved PFS compared to the placebo group among the patients in the unstratified HRD-negative tumours (hazard ratio (HR) 0.68 (0.54–0.85)).^23^ However, the lack of an olaparib/bevacizumab only arm, limited the ability to determine any additional benefit conferred by durvalumab. The recent PFS results from the ATHENA-COMBO (NCT03522246) study failed to demonstrate a benefit from adding nivolumab to rucaparib as maintenance therapy in newly diagnosed advanced high-grade OC patients.^24^ PARPi and bevacizumab monotherapy are approved for OC maintenance in both first and second-line settings, but selecting the optimal sequence is challenging due to reimbursement restrictions with bevacizumab and the licencing restrictions with repeat administration of either therapy following progressive disease (PD).^25^ Tumour angiogenic profiling and selecting appropriate biomarkers could be a future step towards optimising patient selection for bevacizumab therapy.^26^ Furthermore, a positive or negative ‘cut-off’ in HRD tests, each of which uses different techniques to determine HRD, should not be considered as a categoric variable. Rather, it is a probability that the tumour displays a degree of HRD that might benefit from a PARPi. It is known that sensitivity to PARPi correlates with tumour response sensitivity to platinum therapy.^27^ Thus, patients with a tumour that responds well to PBC with a borderline positive HRD test may benefit from a PARPi.

The results of trials with PARPi in the first-line setting and in platinum-sensitive recurrent disease are shown in Table 1. Current licensing by the European Medicines Agency (EMA) and Food and Drug Administration (FDA) differs, in that approval for PARPi maintenance therapy following a response to PBC for recurrent non-BRCAm tumours has been withdrawn by the FDA due to concerns about the OS results. However, the EMA continues to approve olaparib, niraparib or rucaparib in patients responding to PBC for recurrent OC, irrespective of the biomarker status of the tumour. Recommendations for the time to start PARPi after chemotherapy vary, largely because of differences in PARPi trial design. It should be within 8 weeks (olaparib, rucaparib) or 12 weeks (niraparib) after the last dose of chemotherapy. In practice, PARPi should be started when haematological recovery has occurred after chemotherapy, particularly anaemia, to avoid an early pause or dose reduction of PARPi due to their effect on red cell production.

**Table 1.** Summary of PARPi maintenance trials (A) first-line and (B) platinum-sensitive relapse. Phase III study results that have led to PARPi licences.

Study (NCT identifier)	Treatment	Patient population	HR for PFS (95% CI)	Median PFS: PARPi (months)	Median PFS: placebo/control (months)	Median OS: PARPi in months/(rate in %)	Median OS: Placebo or control/(rate in %)	HR for OS (95% CI)
(A)								
SOLO1 (NCT01844986)^1,14,21^	Olaparib vs placebo	BRCAm	0.30 (0.23–0.41)	56.0 (at 5 years)	13.8 (at 5 years)	NR (67% at 7 years)	75.2 (46.5% at 7 years)	0.55 (0.40–0.76)
PRIMA (NCT02655016)^2,13,15^	Niraparib vs placebo	HRD positive	0.43 (0.31–0.59)	21.9 (24.5 at 6 years)	10.4 (11.2 at 6 years)	(55% at 5 years)	(56% at 5 years)	
		BRCAm, HRD positive	0.40 (0.27–0.62)	30.1 (at 6 years)	11.5 (at 6 years)			0.94 (0.63–1.41)
		BRCAwt, HRD positive	0.50 (0.31–0.83)	19.4 (at 6 years)	10.4 (at 6 years)			0.97 (0.62–1.53)
		HRD negative/ unknown	0.68 (0.49–0.94)	8.1 (8.4 at 6 years)	5.4 (5.4 at 6 years)	(29% at 5 years)	(29% at 5 years)	1.01 (0.80–1.27)
PAOLA-1 (NCT02477644)^12,22^	Olaparib + bevacizumab vs placebo + bevacizumab	HRD positive (incl. BRCAm)	0.33 (0.25–0.45)	37.2 (46.8 at 5 years)	17.7 (17.6 at 5 years)	75.2 (65.5% at 5 years)	57.3 (48.4% at 5 years)	0.62 (0.45–0.85)
		BRCAm	0.31 (0.20–0.47)	37.2	21.7	75.2 (73.2% at 5 years)	66.9 (53.8% at 5 years)	0.60 (0.39–0.93)
		BRCAwt, HRD positive	0.43 (0.28–0.66)	28.1	16.6	NR (54.7% at 5 years)	52.0 (44.2% at 5 years)	0.71 (0.45–1.13)
		HRD negative	1.00 (0.75–1.35)	16.6	16.2	36.8 (25.7% at 5 years)	40.4 (32.3% at 5 years)	1.19 (0.88–1.63)
VELIA (NCT02470585)^28^	Carboplatin/ paclitaxel plus, either: (1) veliparib 150 mg then veliparib 400 mg maintenance OR: (2) veliparib 150 mg then placebo maintenance OR: (3) placebo then placebo maintenance	BRCAm	0.44 (0.28–0.68)	34.7	22.0	Data not mature	Data not mature	Data not mature
		HRD positive	0.57 (0.43–0.76)	31.9	20.5	Data not mature	Data not mature	Data not mature
PRIME^29^	Niraparib vs placebo	BRCAm	0.40 (0.23-0.68)	NR	10.8	Data not mature	Data not mature	Data not mature
		BRCAwt	0.48 (0.34-0.67)	19.3	8.3	Data not mature	Data not mature	Data not mature
DUO-O (NCT03737643)^23^	Chemotherapy + bevacizumab (B) ± durvalumab (D) then maintenance (mtx) B ± D ± Olaparib (O)	HRD positive	Mtx B + D: HR vs control, 0.83; (0.60–1.14). Mtx B + D + O: HR vs control, 0.49; (0.34–0.69).	Mtx B: 23.0 Mtx B + D: 24.4 Mtx B + D + O: 37.3		Data not mature	Data not mature	Data not mature
		HRD negative		HR 0.68, (0.54–0.86)		Data not mature	Data not mature	Data not mature
(B)								
SOLO2 (NCT01874353)^4,10^	Olaparib vs placebo	BRCAm	0.30 (0.22–0.41)	19.1	5.5	51.7	38.8	0.74 (0.54–1.00)
SOLO3 (NCT02282020)^30,31^	Olaparib vs physician’s choice of single-agent non-platinum chemotherapy	BRCAm	0.62 (0.43–0.91)	13.4	9.2	34.9	32.9	1.07 (0.76–1.49)
NOVA (NCT01847274)^5,32^	Niraparib vs placebo	BRCAm	0.27 (0.17–0.41)	21.0	5.5	Data not mature	Data not mature	Data not mature
		BRCAwt, HRD positive	0.38 (0.24–0.59)	12.9	3.8	Data not mature	Data not mature	Data not mature
		HRD negative	0.58 (0.36–0.92)	9.3	3.9	Data not mature	Data not mature	Data not mature
ARIEL3 (NCT01968213)^11,19^	Rucaparib vs placebo	BRCAm	0.23 (0.16–0.34)	16.6	5.4	34.9	32.9	1.07 (0.76–1.49)
						45.9 (at 6 years)	47.8 (at 6 years)	0.832 (0.581–1.192)
		HRD (BRCAm or BRCAwt/LOH high)	0.32 (0.24–0.42)	13.6	5.4	40.5 (at 6 years)	47.8 (at 6 years)	1.005 (0.766–1.320)
		BRCAwt/LOH high	0.44 (0.29–0.66)	9.7	5.4			
		BRCAwt/LOH low	0.58 (0.40–0.85)	6.7	5.4			

BRCAm, BRCA mutated; BRCAwt, BRCA wild-type; CI, confidence interval; HR, hazard ratio; HRD, homologous recombination deficiency; LOH, loss of heterozygosity; NR, Not Recorded; OS, overall survival; PARPi, poly (adenosine diphosphate-ribose) polymerase inhibitors; PFS, progression-free survival.

#### Duration

##### First-line maintenance

Defining the optimal length of treatment with PARPi ensures that patients are not over-treated and subjected to unnecessary toxicity, both whilst on therapy and in the longer term. Other considerations include financial costs to health care systems and the risk of driving resistance to future therapy.^33^ These risks must be balanced with an increased potential of progression with undertreatment. Both the FDA and EMA have approved niraparib in all patients who display a response to PBC after front-line treatment and olaparib (either alone in BRCAm tumours, or in combination with bevacizumab for BRCAm or HRD-positive tumours).^7–9,34,35^

Patients in response to front-line treatment are given PARPi maintenance (or placebo) for a set length of time, or until disease progression: niraparib for 3 years, olaparib, alone or in combination with bevacizumab for 2 years or rucaparib for 2 years. Bevacizumab was given for 15 months. Treatment was allowed beyond the 2 or 3 years if there was evidence of ongoing benefit in patients with residual disease. For example, at the time of final data cut-off for the PRIMA trial, almost 6 years after the last patient was randomised, 27 patients were still taking niraparib.^15^ The recommendations for treatment duration are informed by their respective clinical trial designs, without a priori scientific data to support these specific durations of treatment, other than noting that the greatest risk period for recurrence is within 2–3 years after primary therapy.^36,37^ In the SOLO1 trial, which has the longest follow-up, an exploratory analysis of the time to the first subsequent treatment shows that in approximately half the patients with a recurrence, relapse occurred after 2 years with very few relapses occurring after 4 years (2 years post-PARPi completion).^14^ Whether this can be reduced by extending duration of PARPi therapy is unclear. In the PRIMA trial, where niraparib was given for 3 years, relapses were seen between 24 and 36 months, albeit at a slow rate. Relapse continued beyond 36 months, but the rate of relapse remained low.^15^

Late toxicity following PARPi therapy needs to be considered. The rate of myelodysplastic syndrome (MDS)/acute myeloid leukaemia (AML) was low (0.2%–1.1%) in all front-line trials (Table 4) and as expected, slightly higher among patients with a germline BRCA mutation (gBRCAm). Longer follow-up at the time of OS analysis does not demonstrate a significant increase in the number of patients with MDS/AML. Also, the rates of AML/MDS do not appear to differ between studies with different PARPi duration in the first-line setting.

Thus, uncertainty remains about the optimum length of treatment in the front-line setting. Any benefit from prolonging therapy needs to be balanced against the risk of inducing resistance to subsequent platinum-based treatment. In the front-line trials, it is difficult to know whether PARPi increased resistance to subsequent platinum therapy. The little available data related to this come from the exploratory analysis in the PAOLA-1 study, showing that patients relapsing after the completion of olaparib have a similar PFS to control arm patients when subsequently re-treated with platinum-based therapy. Those patients relapsing on olaparib have a short PFS.^38^ Nevertheless, the potential for PARPi to generate platinum resistance remains an important area of research (see Section ‘Treatment following PARPi’).

##### Recurrent setting

Whilst there has been a significant shift towards PARPi use in the front-line setting, there remain patients who have not received PARPi as first-line treatment and are eligible to receive PARPi following PBC for recurrent OC (Table 1). In addition to a documented response to PBC, PARPi are now considered for patients without residual disease after secondary cytoreductive surgery and subsequent PBC. Data on the role of PARPi in this setting are missing; for example, the DESKTOP III study of secondary cytoreductive surgery followed by chemotherapy was completed before PARPi were widely used.^39^ Using a PARPi following cytoreductive surgery opens the possibility of reducing chemotherapy post-surgery. In the NEO phase II window of opportunity study (NCT02489006), olaparib was given for 6 ± 2 weeks prior to secondary cytoreductive surgery in PARPi-naïve patients with PSROC. Following surgery, patients were randomised to either adjuvant olaparib ± chemotherapy to assess the potential for de-escalation therapy. Olaparib monotherapy was as effective as chemotherapy followed by olaparib with no difference in the 3-year PFS and OS rates, and was less toxic, demonstrating the feasibility for a chemotherapy-free approach in this selected population.^40^ The key difference between PARPi use in recurrent disease and front-line therapy is the duration of therapy. In the recurrent disease setting, PARPi are indicated until progression or toxicity. Patients may be in complete remission following chemotherapy and free from progression for months or years. The duration of remission is generally longer in patients with a BRCAm, but all studies have reported a group of long-term responders remaining without disease for 4 or more years.^14,19,41,42^ It remains unclear how long a patient should remain on a PARPi for recurrent OC. Guidance on the appropriate length of treatment is not available as there is a lack of evidence surrounding treatment duration. Concern about the risk of developing MDS/AML or future drug resistance needs to be balanced against the risk of recurrence on stopping treatment. Of note, without any PARPi, the PFS with maintenance placebo was around 5.5 months in all trials. A recent survey from 210 physicians in 26 countries reports a diverse practice of continuing PARPi or stopping after a finite period.^43^ There is now an ongoing retrospective international audit for outcome and toxicity of patients receiving a PARPi for 5 or more years.

#### Dosing

Treatment interruption and dose reductions were a feature of all PARPi studies. For example, in the phase III trials of olaparib, niraparib or rucaparib in recurrent disease, dose reduction occurred in 25%–66%.^4,5,11^ The greatest reduction due to early myelotoxicity was seen in the NOVA trial and a subsequent analysis (RADAR trial) identified patients with a baseline body weight of <77 kg or baseline platelets of <150 × 10^9^/L as being at greater risk of haematological toxicity. In an analysis of patients treated with 300 or 200 mg niraparib/day, patients on the lower dose of niraparib did not have a worse outcome.^44^ Dose-adjusted treatment was introduced in subsequent niraparib trials (PRIMA,^2^ NORA,^44^ and PRIME^29^). Flexibility in relation to dose is important. There are fewer dosing options with niraparib where the starting dose is 200 mg, with only one dose reduction to 100 mg daily. This contrasts with rucaparib where 600 mg bid is the starting dose with a possible three-step dose reduction to 300 mg bid. For olaparib tablets, 300 mg is the starting dose with options to reduce in increments of 50 mg, usually stopping at 200 mg bid. It is unusual to reduce the dose of olaparib to 100 mg bid. Tolerance of treatment at lower dose needs be weighed up against discontinuation of treatment due to toxicity. However, little information exists about the effect of dose reduction on disease control in either recurrent OC or in front-line maintenance treatment.

##### Intermittent dosing

Currently, all PARPi are licenced for continuous, daily dosing and this schedule has been informed by clinical studies.^45,46^ Pre-clinical work demonstrated some delay in tumour progression with intermittent scheduling of PARPi in BRCA-1 deficient mice.^47^ An adaptive dosing approach, which adjusts PARPi dosing based on tumour dynamics, has been examined in OC cell populations using mathematical modelling and in vitro experiments. This showed that adaptive dosing strategies could optimise PARPi maintenance therapy, offering a more personalised and potentially less toxic treatment option for patients.^48^ Clinical studies have explored intermittent dosing of PARPi as monotherapy^49^ and in combination with other agents.^50,51^ It is unclear how these could affect development of PARPi resistance or subsequent platinum resistance following PARPi therapy. How best to select the patient population who may benefit from these approaches also remains unclear.

#### PARPi as chemotherapy sparing neoadjuvant agent

It is unknown whether there is a role for PARPi in the neoadjuvant setting to enhance surgical outcomes and/or reduce chemotherapy requirements.^52^ For neoadjuvant PARPi to be beneficial, it would need to increase the rates of complete surgical cytoreduction. There are several ongoing trials, exploring the feasibility and efficacy of using neoadjuvant PARPi summarised in Table 2. In the neoadjuvant NANT study with niraparib, 30 out of 48 patients achieved a partial response (PR) and 12 had stable disease (SD), which resulted in an overall response rate (ORR) of 62.5% and a disease control rate (DCR) of 87.5%. The BRCAm cohort did better (ORR (77.3%) and DCR (100.0%)). Forty patients underwent interval cytoreductive surgery, 80% achieved complete macroscopic resection (no residual disease) and 95.0% optimal cytoreduction.^53^ The NOW study evaluated the feasibility of two cycles of neoadjuvant olaparib in 15 patients; 13 patients (87%) underwent cytoreductive surgery immediately after olaparib and 2 (13%) received chemotherapy prior to surgery, of whom 1 was not fit for subsequent surgery. Fourteen (100%) patients had optimal cytoreductive surgery: 12 (86%) had a complete gross resection, and 1 patient (8%) had a pathologic complete response.^54^ The OLAPem study evaluated neoadjuvant olaparib as monotherapy^55^ and in combination with pembrolizumab.^56^ Twenty patients were enrolled in the immunotherapy combination cohort, 17 of whom underwent tumour cytoreductive surgery (2 immediately, while 15 received preoperative chemotherapy), which suggests cytoreductive surgery was not feasible following the study treatment in the majority of patients.^56^ The NUVOLA study evaluated the addition of olaparib to neoadjuvant chemotherapy and enrolled 35 patients. Grade 3 or higher haematological treatment-related adverse events were very common (83.3%) and only 11.4% of cases achieved a pathological complete response, meaning the trial did not meet its primary endpoint.^57^ These results, together with those awaited from other studies (Table 2), will inform whether there is a role for PARPi in this context as a chemotherapy sparing agent in selected patient groups. A high tumour response rate is seen with drugs such as carboplatin, which is well-tolerated. PARPi are unlikely to replace platinum, and compounding of myelotoxicity with PARPi and carboplatin is a potential issue. For this combination to be worthwhile response rates would need to be considerably greater, or lead to a sparing of other toxic drugs, such as paclitaxel in patients with large volume disease, who need a rapid reduction in tumour burden for symptomatic improvement.

**Table 2.** Studies evaluating neoadjuvant PARPi.

Study (NCT trial identifier)	Type of trial	Treatment	Population	BRCA status	Primary endpoint
NOW (NCT03943173)	Single-arm, open label, phase I	⩽2 cycles olaparib, then tumour reductive surgery (if no PD), or chemotherapy (if PD or surgery unsuitable)	Advanced high-grade epithelial OC	Germline mutation in BRCA, RAD51C/D or PALB2	Feasibility
NUVOLA (NCT04261465)	Multi-centre, single arm, phase II	3 cycles neoadjuvant chemotherapy with weekly olaparib (days 1–3) and subsequent interval cytoreductive surgery and adjuvant chemotherapy	Advanced primary OC not suitable for primary cytoreductive surgery	BRCAm/pathogenic variants	Pathological complete response rate
IMPACT (NCT03378297)	Single centre, single arm, window-of-opportunity phase I	Olaparib for 10–14 days before surgery	Advanced high-grade serous OC	Regardless of mutational status	Change in biomarker expression
OLAPem (NCT04417192)	Multi-centre, single-arm, open-label, phase II	Olaparib ± pembrolizumab for two cycles before surgery	Advanced high-grade serous or grade 3 endometrioid, epithelial OC	HRD positive	ORR
NANT (NCT04507841)	Multi-centre single-arm, open label, phase II	Two cycles niraparib before surgery, followed by carboplatin/paclitaxel in patients with CR/PR or SD	High-grade serous or endometrioid OC	HRD positive	ORR; complete macroscopic clearance rate
OPAL-C (NCT03574779)	Multi-centre, randomised, open-label, phase II	Three cycles niraparib or carboplatin/paclitaxel before interval cytoreductive surgery. Followed by three cycles of carboplatin and paclitaxel (± bevacizumab) and maintenance niraparib	Newly diagnosed advanced OC	HRD positive	Pre-interval cytoreductive surgery ORR

BRCAm, Breast Cancer Susceptibility Gene mutation; CR, complete response; HRD, homologous recombination deficient; OC, ovarian cancer; ORR, overall response rate; PALB2, partner and localiser of BRCA2; PARPi, poly (adenosine diphosphate-ribose) polymerase inhibitors; PD, progressive disease; PR, partial response; SD, stable disease.

### Treatment following PARPi

PARPi resistance with disease progression is common in both front-line and recurrent therapy. Resistance may be primary, with drug-resistant clones present at the outset of treatment or acquired, driven by use of the PARPi.^58^ The mechanisms underlying resistance are complex and not fully understood.^58,59^ Those that restore homologous recombination repair (HRR), include reversion mutations, which can reinstate the function of BRCA1/2 or other HRR genes,^60^ and epigenetic changes, which can lead to re-expression of previously silenced HRR genes.^60^ Other mechanisms work by decreasing PARP trapping, via mutations in PARP1 (e.g. R591C), which reduce PARP1 trapping on DNA and loss of PAR glycohydrolase, leading to PAR accumulation.^61^ Drug efflux can be mediated by upregulation of ABCB 1a/1b genes and enhanced expression of P-glycoprotein efflux pumps.^62^ Replication fork stabilisation enhances protection against replication fork degradation through various mechanisms, including activation of Ataxia telangiectasia and Rad3-related protein serine/threonine kinase (ATR)/CHK1/WEE1 signalling.^63^ It is important to understand how the different mechanisms of resistance to PARPi affect subsequent chemotherapy treatments.^38^

Data on chemotherapy use post PARPi have not clarified the optimum chemotherapy sequence.^64–66^ PBC is often used, if the platinum-free interval (PFI) is at least 6 months, but as PARPi and platinum agents are both dependent on the DNA damage response (DDR) pathway it is not surprising that in some cases, cross-resistance mechanisms occur.^52^ A post hoc analysis of the SOLO2 trial showed that among BRCAm patients who received PBC as their first subsequent treatment after progression, the time to second progression was longer in the placebo arm compared with the olaparib arm (14.3 vs 7.0 months),^16^ suggesting that PARPi affect subsequent benefit from PBC.^16^ In the ARIEL4 trial, where rucaparib was compared to investigator-choice chemotherapy in patients with recurrent BRCAm OC, patients receiving chemotherapy were allowed to cross over to rucaparib at the point of progression^67^ and the updated OS results did not show an improvement with rucaparib; median OS was 19.4 months in the rucaparib group compared to 25.4 months in the chemotherapy group (HR = 1.31, 95% confidence interval (CI) = 1.00–1.73, *p* = 0.0507). Patients experienced longer PFS with rucaparib if they received paclitaxel first followed by rucaparib, rather than the converse. However, it should be noted that fewer patients on the rucaparib arm received any additional systemic treatment, and this could have contributed to the lower OS seen with rucaparib in this trial. Similarly in the SOLO3 study comparing olaparib with non-PBC in patients with gBRCA PSROC, there was a numerical detriment in the median OS in the olaparib arm compared to chemotherapy (34.9 vs 32.9 months; HR 1.07, *p* = 0.714, respectively).^30^ Although both trials met their primary endpoint with an improvement in PFS, the unfavourable results in the underpowered secondary endpoint of OS led to questions about the use of PARPi as monotherapy in patients with recurrent BRCAm OC, leading to a withdrawal in the use of these drugs as monotherapy in this setting.^68,69^

Whilst most of the information on subsequent chemotherapy use after PARPi has come from trials in recurrent OC, data are now emerging on subsequent treatment after front-line use of PARPi. In an exploratory post hoc analysis of PAOLA-1, the outcome of patients who received PBC following progression on or after first-line maintenance with olaparib was different.^38^ The time from first to second subsequent treatment was similar in the control and olaparib arms among patients relapsing after the completion of olaparib therapy. However, those relapsing during olaparib had a shorter interval between treatments.^38^ As data continue to emerge about treatment post-PARPi, it becomes increasingly important to understand the mechanism underlying progression and why some patients respond, while others are resistant to subsequent therapies. This is best achieved through translation studies.^70^

### PARPi re-challenge

There is relatively little information about PARPi rechallenge, regardless of whether disease relapse occurs whilst on therapy or following treatment. There are many ongoing studies (Table 3) to explore this, using PARPi alone or in combination with other drugs (see Section ‘Combination approaches towards tackling resistance’). Establishing whether there is a role for PARPi rechallenge is important, since other than bevacizumab, there are no currently available options for maintenance therapy after chemotherapy for relapsed disease. The phase IIIb OReO/ENGOT-ov38 NCT03106987 trial was the first randomised trial to formally investigate whether retreatment with PARPi after chemotherapy for PSROC was beneficial. Most patients previously received a prior PARPi in the recurrent setting (i.e. second or third line) and therefore had progressed while on the drug. There was a modest, yet statistically significant improvement in PFS irrespective of BRCA status, however, the results were not deemed to be clinically meaningful. The results are summarised in Table 3. The Kaplan–Meier curves showed that 40%–50% of patients might display olaparib resistance, whereas 10%–15% of patients might achieve prolonged tumour control (>18 months),^71^ suggesting that prior PARPi exposure does not indicate complete resistance to therapy. As stated above, an exploratory post hoc analysis of the PAOLA-1 study reported the outcome of patients relapsing after completion of olaparib who were treated with chemotherapy and subsequently a PARPi. These patients had a longer median time from first to second subsequent therapy than those who received platinum alone.^72^

**Table 3.** Studies evaluating PARPi rechallenge.

Name of trial	Phase	Population	Treatment	Results
OReO/ENGOT-ov38 (NCT03106987)^71^	III	PSROC, 1 prior line PARPi	Olaparib or placebo	Median PFS (months); olaparib vs placebo. BRCAm: 4.3 vs 2.8; (HR 0.57, 95% CI 0.37–0.87; *p* = 0.022). BRCAwt: 5.3 vs 2.8; (HR 0.43, 95% CI 0.26–0.71; *p* = 0.002).
DUETTE (NCT04239014)	II	PSROC, 1 prior line PARPi	Ceralasertib (VEGFi) ± olaparib or placebo	Study terminated
MOLTO (NCT02855697)^75^	II	PSROC; PARPi-naïve and prior olaparib cohorts	Maintenance olaparib ± cediranib (VEGFi)	Duration of first and second olaparib maintenance: (12.1 vs 4.4 months; *p* < 0.001)
QUADRA (NCT02354586)^73^	II	PSROC, ⩾3 prior lines of chemotherapy	Niraparib 300 mg	ORR in prior niraparib subgroup: 6%
DDRiver EOC 302 (NCT06433219)	II	PSROC, PD during PARPi	Tuvusertib (ATRi) + lartesertib (ATMi) or Tuvusertib + niraparib	Recruiting
EVOLVE (NCT02681237)^79^	II	Platinum sensitive/resistant/PD on chemo and post PARPi progression	Cediranib + olaparib	(a) ORR (%) (b) 16-Week PFS rate (%) platinum-sensitive: (a) 0 (b) 55 platinum-resistant: (a) 20 (b) 50 PD on chemo: (a) 8 (b) 39
NIRVANA-R (NCT04734665)^80^	II	PSROC, ⩾2 prior lines chemotherapy, with CR/PR to last platinum regimen	Niraparib and bevacizumab maintenance	Met efficacy to proceed to the second stage
CAPRI (NCT03462342)^81^	II	PARPi after 1st line chemo for ⩾12 months OR after >1 line for ⩾6 months	Olaparib and ceralasertib	ORR: 50% (95% CI, 0.15–0.72). Median treatment: 8 cycles (range 4–23+)

ATMi, Ataxia telangiectasia mutated inhibitor; ATRi, Ataxia telangiectasia and Rad3-related inhibitor; BRCAm, BRCA mutated; BRCAwt, BRCA wild-type; CI, confidence interval; CR, complete response; HR, hazard ratio; OR, odds ratio; ORR, objective response rate; PARPi, poly (adenosine diphosphate-ribose) polymerase inhibitors; PD, progressive disease; PFS, progression-free survival; PR, partial response; PSROC, platinum-sensitive recurrent ovarian cancer; VEGFi, vascular endothelial growth factor inhibitor.

The phase II QUADRA (NCT02354586) trial evaluated niraparib monotherapy in patients with PSROC who had received at least three lines of therapy. A subgroup analysis was performed on 37 patients who received niraparib following prior PARPi; the ORR was 6%, one patient had a confirmed PR and the clinical benefit rate at 16 weeks was 20%, indicating some disease control.^73^ The randomised phase II Duette study (NCT04239014) assessed the efficacy of the ATR inhibitor, ceralasertib with olaparib, and olaparib monotherapy, compared with placebo, as second maintenance therapy in patients with PSROC, who had received previous maintenance PARPi.^74^ However, the trial was terminated following closure of the VIOLETTE study (NCT03330847), investigating the combination of ceralasertib and olaparib in triple-negative breast cancer, due to poor efficacy (NCT04239014). The single-arm phase II MOLTO trial explored the feasibility of a second maintenance course of olaparib in participants diagnosed with gBRCAm, PSROC with either olaparib alone if they had a PFI of at least 6 months or with maintenance olaparib and cediranib if they achieved a PFI of less than 6 months duration. A second course of olaparib was safe and generally well tolerated but was only modestly effective.^75^ Other information emerging is derived from small-scale single-centre^76,77^ and multicentre^78^ institutional retrospective reviews. The indications are that re-treatment with a PARPi is feasible and may be beneficial in some patients. It is an area that needs formal investigation, particularly as there are very few options for maintenance therapy in patients previously treated with a PARPi.

### Management of low-volume recurrence or oligometastatic disease

Recurrence during PARPi therapy may be low volume, or oligometastatic (⩽5 metastases). Whether this is due to a more intensive image-guided follow-up of patients on PARPi or the emergence of sub-clonal resistance mutations is unclear.^82^ Strategies to manage these types of progression are evolving although they are not evidence-based. The aim is to delay the time before there is a need for further chemotherapy. An ongoing phase III trial (NCT05607329) is investigating whether careful selection of patients who progress on PARPi maintenance benefit from cytoreductive surgery; 400 patients with platinum-sensitive OC who have recurred following at least 6 months of PARPi maintenance are randomised between secondary cytoreductive surgery followed by PBC or chemotherapy alone.^83^ An additional consideration for patients with oligometastatic recurrence on PARPi, who undergo locoregional treatment (surgery, ablation or radiotherapy) is whether patients should continue PARPi. Small retrospective cohort data suggest that patients with oligometastatic progression on PARPi may continue to benefit from PARPi maintenance following locoregional treatment.^84,85^ For example, one study reported the outcome of 186 patients who continued with PARPi maintenance until further progression, after surgery or stereotactic body radiotherapy (SBRT). The median treatment-free interval was 6 months for those treated with surgery and 10 months with SBRT.^84^ Another retrospective study evaluated the efficacy of PARPi continuation in 74 patients after local treatment for oligometastatic progression; median PFS following local therapy was 11.5 months (95% CI 7.4; 17.2) and the 1-year OS rate was 90.7% (95% CI 79.1; 96.0).^85^ Prospective randomised trials are needed, as the reported studies were retrospective, making it difficult to ascertain the contribution of the PARPi and local treatment. One such study is the phase II SOPRANO trial (NCT05990192) exploring the use of SBRT in two cohorts of patients with oligometastatic progression on PARPi; one receiving SBRT alone and the other treated with SBRT followed by niraparib (NCT05990192).

### Combination approaches towards tackling resistance

Building on our understanding of the mode of action of PARPi and potential resistance mechanisms, other molecules which inhibit the DDR pathway and DNA repair are under evaluation. The main direction of this research is to develop small molecules,^86^ such as cell-cycle checkpoint kinase inhibitors, for example, ATR,^81,87,88^ Ataxia telangiectasia mutated,^89^ that could be used to overcome complete or partial resistance to PARPi. Whilst these drugs may be given as monotherapy, there is interest in combining them with PARPi. Other combination strategies include PARPi with Checkpoint kinase 1 (CHK1)^90^ and WEE1^91^ targeting drugs. Targeting alternative repair pathways, such as repair of DNA double-strand breaks through microhomology-mediated end joining, by POLθ (polymerase theta), a DNA polymerase and DNA helicase fusion protein, is also being evaluated.^92^ This provides an alternative DDR pathway when homologous recombination is compromised and so POLθ can help cancer cells survive despite PARP1 inhibition, thus reducing the efficacy of PARPi. Inhibiting POLθ to force cancers to rely on defective homologous recombination and accumulate DNA damage, is an emerging therapeutic target either alone or in conjunction with PARPi^93,94^ and some studies have reported specific Polθ inhibitors with in vivo efficacy, which may offer a promising therapy to bypass PARPi resistance in HRD-positive tumours.^95,96^

The interaction between DDR and the immune system provides the basis to combine PARPi with ICIs in patients with HRD-positive tumours, to transform ‘cold’ tumours into ‘hot’ tumours and improve the response to immunotherapy. This synergy may be mediated through several mechanisms, including an increase in neoantigen burden.^97,98^ Conversely, ICIs can increase PARPi sensitivity by priming and activating immune cells to promote an immune cytotoxic effect.^99^ PARPi can also modify the microenvironment, thereby complementing the activity of PD-1/PD-L1 blockade.^100^ Cyclic guanosine monophosphate–adenosine monophosphate synthase (cGAS)–stimulator of interferon genes (STING) pathway-mediated immune priming is another mechanism underlying this synergy between PARPi and ICI, via cGAS binding^101^ and subsequent tumour infiltration by immune cells.^101,102^

Several studies exploring the combination of PARPi and ICIs^103–106^ are in progress and will add to the aforementioned DUO-O and ATHENA COMBO data. For example, the phase II MEDIOLA study demonstrated promising efficacy results with olaparib, durvalumab and bevacizumab therapy in non-gBRCAm PSROC; the DCR at 24 weeks was 28.1% (90% CI, 15.5–43.9) in the doublet cohort (olaparib and durvalumab) and 74.2% (90% CI, 58.2–86.5) in the triplet cohort, and DCR at 56 weeks was 9.4% (90% CI, 2.6–22.5) and 38.7% (90% CI, 24.1–55.0), respectively.^105^ The results of two other large-scale phase III trials are awaited. These are the ENGOT-Ov44/FIRST NCT03602859 trial combining dostarlimab and niraparib and the MK-7339-001/KEYLYNK-001/ENGOT-ov43/GOG-3036 study (NCT03740165) combining olaparib and pembrolizumab.

Current PARPi block PARP1 and PARP2 enzymes^107,108^ and whilst effective, the dose is limited by toxicity, particularly haematological side effects, which is enhanced by inhibition of PARP2.^109^ Loss of PARP1 is a major driver of synthetic lethality with HRD^110,111^ and PARP1 selective agents are being developed as more potent inhibitors with less toxicity, due to fewer off-target effects.^112^ The phase I/IIa PETRA trial (NCT04644068) is evaluating the next-generation PARP1-selective inhibitor AZD5305 (saruparib) in patients with BRCAm, Partner and localiser of BRCA2 (PALB2) or RAD51C/D mutations across several tumour types, including progressive advanced/metastatic OC. Initial results show that AZD5305 achieved higher fold coverage over the target effective concentration compared to first-generation PARPi and has a favourable safety profile.^113^

### Follow-up

#### Monitoring for progression

No consensus guidelines exist for how to follow up patients on PARPi, particularly the frequency of CA-125 testing and imaging. The Gynaecological Cancer InterGroup definition of CA-125 progression^114^ is most commonly used in clinical practice, but it has recently been shown that during PARPi maintenance, there is discordance between CA-125 and RECIST PD.^115^ A post hoc analysis of the SOLO2 trial^116^ and a recent meta-analysis of four PARPi versus placebo trials^117^ found that nearly half of patients without CA-125-defined progression had RECIST PD on surveillance CT imaging, with mostly normal CA-125 values. The authors concluded that regular CT imaging should be considered as part of surveillance on PARPi, especially for those with a normal CA-125 at the start of maintenance therapy and during treatment.^117^

#### Monitoring for myeloid toxicity

Secondary myeloid neoplasms,^118^ including AML and MDS, are associated with PARPi treatment.^119^ Whilst the exact aetiology is unknown, it is possible that PARPi and chemotherapy exert selective pressure and cause expansion of DDR-altered clonal haematopoiesis, which increases the risk of myeloid neoplasms.^120^ Multiple risk factors appear to contribute, including cumulative platinum exposure and genetic predisposition.^121^ More information is emerging as long-term PARPi trial data are published. These suggest that the myeloid neoplasm rates across some trials are higher than reported in the original preliminary data (Table 4); for example, after 6 years of follow-up in SOLO2, there was a 4-fold increase in myeloid malignancy rates from 2.1% to 8%^10^ and in the NOVA trial, a preliminary analysis revealed a 1.3% increased risk, which increased to 2.1% increase at 5 years.^32^ The risk is higher in patients with gBRCAm and seems also to be greater when PARPi are used to treat recurrent OC. This may be partly due to the duration of treatment, although on average this is less than in the front-line setting, or due to the compounding effect of prior cytotoxic chemotherapy^120^ (Table 4). A meta-analysis demonstrated an increased risk of MDS/AML across PARPi^119^ with one study suggesting a greater level in patients receiving front-line PARPi.^33^ The rate of second primary malignancies may also be increased by PARPi therapy although the magnitude is unknown. A meta-analysis of 23 placebo RCTs involving nearly 9000 patients found nearly identical rates of secondary primary malignancies in both the PARPi and placebo arms.^122^

**Table 4.** Rates of myeloid neoplasms as reported from pivotal randomised controlled trials of patients with advanced ovarian cancer receiving PARPi.

Setting	Trial (PARP inhibitor)	Primary analysis	Long-term follow-up
First-line maintenance	SOLO1 (olaparib vs placebo)	1% vs 0%^1^	7 years: 1.5% vs 0.8%^14^
	PRIMA (niraparib vs placebo)	0.2% vs 0%^2^	3.5 years: 1.2% vs 1.2%^13^
	ATHENA-MONO (rucaparib vs placebo)	0.5% vs 0%^20^	No data
	PAOLA-1 (olaparib + bevacizumab vs bevacizumab + placebo)	1.1% vs 0.4%^22^	5 years: 1.7% vs 2.2%^12^
Recurrent maintenance	SOLO2 (olaparib vs placebo)	2.1% vs 4%^4^	6 years: 8% vs 4%^10^
NOVA (niraparib vs placebo)	1.4% vs 1.1%^5^	5.5 years: 3.5% vs 1.7%^32^	ARIEL3 (rucaparib vs placebo)
1% vs 0%^11^	6 years: 3.8% vs 3.2%^19^		

Source: Adapted from Carus et al.120

PARPi, poly (adenosine diphosphate-ribose) polymerase inhibitors.

Longer term follow-up of current phase III trials and more real-world data are needed to understand the interplay of PBC and genetic factors in driving the incidence of myeloid neoplasms associated with PARPi,^123^ particularly since pharmacovigilance reporting data suggest a higher rate of myeloid neoplasms post PARPi in the real-world setting.^120^ Predictive biomarkers to identify patients at highest risk, tailoring surveillance measures during and following treatment with PARPi are needed. The contribution of BRCA/HRD, the risk profiles of different PARPi together with combination treatments, including the newer selective PARP1 inhibitors and PARPi rechallenge towards the development of myeloid neoplasms also need clarifying.^120^

### Epidemiological factors

Many of the key PARPi studies conducted to date either did not report ethnicity^10^ or recruited a majority Caucasian population,^5,11,124^ which limits applicability to real-world populations.^125^ The phase III NORA study was the first randomised phase III trial of PARPi maintenance therapy conducted in an exclusively Chinese patient population with PSROC. Niraparib maintenance was effective and well tolerated in these patients, regardless of BRCAm status, and prolonged PFS compared with placebo, although no HRD testing was performed.^126^ The findings were in keeping with the ENGOT-OV16/NOVA study with comparable HR for PFS among the BRCAm subgroups and reduction in risk of PD or death for niraparib versus placebo. The NORA study population displayed a relatively high g*BRCA*m rate (37.7%), which may reflect a higher prevalence of gBRCAm in Chinese patients with OC (estimated at 23.1%–28.5%) compared with 11.7%–14.1% in women with OC from Australia and the United States.^126–128^ The PRIME study, a first-line niraparib maintenance study, was also conducted in Chinese patients. This study enrolled patients with less advanced disease than participants in the PRIMA study and included patients with stage 3 disease with complete surgical resection, a population that had been excluded from PRIMA. It demonstrated a significant reduction in the risk of disease progression and met its safety endpoint.^29^ Recently, a phase III study using senaparib versus placebo in Chinese patients as first-line maintenance therapy after PBC in advanced OC, reported significantly improved PFS with senaparib, irrespective of BRCAm status, displayed consistent benefits between homologous recombination subgroups and was well tolerated.^129^ A recent retrospective analysis that included 48 phase II and III trials, found that non-Hispanic black and Hispanic OC patients were significantly underrepresented compared to non-Hispanic white patients (odds ratio (OR) 0.23, 95% CI (0.18–0.29) and OR 0.3, 95% CI (0.25–0.38), respectively, *p* < 0.001).^130^ This is important since the pharmacokinetics of some PARPi are affected by race; for example, talazoparib clearance is 24.7% higher and exposure approximately 20% lower in Asian patients compared with non-Asian patients.^131^ Differences in P-glycoprotein and Breast Cancer Resistance Protein polymorphisms, may contribute to this, with a higher frequency of single nucleotide polymorphisms present in Asian individuals compared with white individuals.^131^ The effect of pharmacogenomic variations on rucaparib pharmacokinetics is less certain due to the unbalanced Asian and Black proportion of patients included in studies,^132^ whilst race does not significantly affect the pharmacokinetics of niraparib.^133^ Furthermore, the frequency of pathogenic variants occurring within OC varies with ethnicity, with BRCA1 reported to occur in 1% of individuals of African descent, 7% in Whites and 16% in Hispanics.^134^ Due to the varying genetics in different racial groups, the results of trials limited to specific racial groups cannot be directly extrapolated to other racial and ethnic populations and more inclusive trial designs are warranted to better represent real-world data.

The influence of age on the efficacy of PARPi is not well described due to the limited inclusion of older adults in clinical trials, leading to a lack of robust data specifically addressing the efficacy of PARPi in the elderly.^135^ Age-related changes in drug metabolism and elimination can affect the pharmacokinetics of PARPi; older adults often have reduced renal and hepatic function, which can alter drug clearance and potentially impact efficacy and toxicity.^136^ The side effect profile in older adults may vary, which can impact dosing and continuity. A literature review found that rates of grade 3 or more lymphopenias were higher in studies including older patients.^137^ Furthermore, the biology of OC can change with age and older patients may have different tumour characteristics compared to younger patients, which could impact efficacy.^138^ Clinical trials dedicated to older patients are needed to help address some of these factors.

## Conclusion

PARPi have led to a major change in the treatment of high-grade OC. All trials have confirmed significant improvements in PFS with maintenance PARPi. However, there remain many unknowns about the activity and use of these drugs that require further study. Some of the key debates about their use and incorporation into different phases of treatment have been highlighted. More precise biomarkers that will predict the outcome of PARPi therapy are needed. Above all, resistance to PARPi is a major obstacle to long-term benefit. DNA repair and the DDR pathways are complex, and the mechanisms underlying resistance, leading to successful repair of DNA damage need to be better understood. There is nevertheless a real benefit to many patients. Longer follow-up of existing randomised controlled trials, and ongoing real-world data reporting will contribute further longer-term data on survival and toxicity. DNA damage and repair remain a key target for therapies in OC, and directing further research to this area will add to the success gained from PARPi therapy.

## Declarations

**Ethics approval and consent to participate:** Not applicable.

**Consent for publication:** Not applicable.

**Availability of data and materials:** Not applicable.

## References

[1] Moore K Colombo N Scambia G , et al. Maintenance olaparib in patients with newly diagnosed advanced ovarian cancer. N Engl J Med 2018; 379: 2495–2505. 30345884 10.1056/NEJMoa1810858

[2] Gonzalez-Martin A Pothuri B Vergote I , et al. Niraparib in patients with newly diagnosed advanced ovarian cancer. N Engl J Med 2019; 381: 2391–2402. 31562799 10.1056/NEJMoa1910962

[3] Monk BJ Coleman RL Fujiwara K , et al. ATHENA (GOG-3020/ENGOT-ov45): a randomized, phase III trial to evaluate rucaparib as monotherapy (ATHENA-MONO) and rucaparib in combination with nivolumab (ATHENA-COMBO) as maintenance treatment following frontline platinum-based chemotherapy in ovarian cancer. Int J Gynecol Cancer 2021; 31: 1589–1594. 34593565 10.1136/ijgc-2021-002933 PMC8666815

[4] Pujade-Lauraine E Ledermann JA Selle F , et al. Olaparib tablets as maintenance therapy in patients with platinum-sensitive, relapsed ovarian cancer and a BRCA1/2 mutation (SOLO2/ENGOT-Ov21): a double-blind, randomised, placebo-controlled, phase 3 trial. Lancet Oncol 2017; 18: 1274–1284. 28754483 10.1016/S1470-2045(17)30469-2

[5] Mirza MR Monk BJ Herrstedt J , et al. Niraparib maintenance therapy in platinum-sensitive, recurrent ovarian cancer. N Engl J Med 2016; 375: 2154–2164. 27717299 10.1056/NEJMoa1611310

[6] Kristeleit R Lisyanskaya A Fedenko A , et al. Rucaparib versus standard-of-care chemotherapy in patients with relapsed ovarian cancer and a deleterious BRCA1 or BRCA2 mutation (ARIEL4): an international, open-label, randomised, phase 3 trial. Lancet Oncol 2022; 23: 465–478. 35298906 10.1016/S1470-2045(22)00122-X

[7] European Medicines Agency (EMA). Lynparza, https://www.ema.europa.eu/en/medicines/human/EPAR/lynparza (2022, accessed 3 March 2024).

[8] European Medicines Agency (EMA). Rubraca, https://www.ema.europa.eu/en/medicines/human/EPAR/rubraca (2022, accessed 12 December 2024).

[9] European Medicines Agency (EMA). Zejula, https://www.ema.europa.eu/en/medicines/human/EPAR/zejula (2022, accessed 12 December 2024).

[10] Poveda A Floquet A Ledermann JA , et al. Olaparib tablets as maintenance therapy in patients with platinum-sensitive relapsed ovarian cancer and a BRCA1/2 mutation (SOLO2/ENGOT-Ov21): a final analysis of a double-blind, randomised, placebo-controlled, phase 3 trial. Lancet Oncol 2021; 22: 620–631. 33743851 10.1016/S1470-2045(21)00073-5

[11] Coleman RL Oza AM Lorusso D , et al. Rucaparib maintenance treatment for recurrent ovarian carcinoma after response to platinum therapy (ARIEL3): a randomised, double-blind, placebo-controlled, phase 3 trial. Lancet 2017; 390: 1949–1961. 28916367 10.1016/S0140-6736(17)32440-6 PMC5901715

[12] Ray-Coquard I Leary A Pignata S , et al. Olaparib plus bevacizumab first-line maintenance in ovarian cancer: final overall survival results from the PAOLA-1/ENGOT-ov25 trial. Ann Oncol 2023; 34: 681–692. 37211045 10.1016/j.annonc.2023.05.005

[13] Gonzalez-Martin A Pothuri B Vergote I , et al. Progression-free survival and safety at 3.5 years of follow-up: results from the randomised phase 3 PRIMA/ENGOT-OV26/GOG-3012 trial of niraparib maintenance treatment in patients with newly diagnosed ovarian cancer. Eur J Cancer 2023; 189: 112908. 37263896 10.1016/j.ejca.2023.04.024

[14] DiSilvestro P Banerjee S Colombo N , et al. Overall survival with maintenance olaparib at a 7-year follow-up in patients with newly diagnosed advanced ovarian cancer and a BRCA mutation: the SOLO1/GOG 3004 trial. J Clin Oncol 2023; 41: 609–617. 36082969 10.1200/JCO.22.01549 PMC9870219

[15] Monk BJ Barretina-Ginesta MP Pothuri B , et al. Niraparib first-line maintenance therapy in patients with newly diagnosed advanced ovarian cancer: final overall survival results from the PRIMA/ENGOT-OV26/GOG-3012 trial. Ann Oncol 2024; 35: 981–992. 39284381 10.1016/j.annonc.2024.08.2241 PMC11934258

[16] Frenel JS Kim JW Aryal N , et al. Efficacy of subsequent chemotherapy for patients with BRCA1/2-mutated recurrent epithelial ovarian cancer progressing on olaparib versus placebo maintenance: post-hoc analyses of the SOLO2/ENGOT Ov-21 trial. Ann Oncol 2022; 33: 1021–1028. 35772665 10.1016/j.annonc.2022.06.011

[17] Wu T Zhang P Wang G. Long-term outcomes in the PRIMA trial: a closer look at progression-free survival and overall survival. Ann Oncol 2025; 36: 340. 39537038 10.1016/j.annonc.2024.11.003

[18] Ledermann JA Harter P Gourley C , et al. Overall survival in patients with platinum-sensitive recurrent serous ovarian cancer receiving olaparib maintenance monotherapy: an updated analysis from a randomised, placebo-controlled, double-blind, phase 2 trial. Lancet Oncol 2016; 17: 1579–1589. 27617661 10.1016/S1470-2045(16)30376-X

[19] Coleman RL Oza A Lorusso D , et al. O003/#557 Overall survival results from ARIEL3: a phase 3 randomized, double-blind study of rucaparib vs placebo following response to platinum-based chemotherapy for recurrent ovarian carcinoma. Int J Gynecol Cancer 2022; 32: A3.2–A.4.

[20] Monk BJ Parkinson C Lim MC , et al. A randomized, phase III trial to evaluate rucaparib monotherapy as maintenance treatment in patients with newly diagnosed ovarian cancer (ATHENA-MONO/GOG-3020/ENGOT-ov45). J Clin Oncol 2022; 40: 3952–3964. 35658487 10.1200/JCO.22.01003 PMC9746782

[21] Banerjee S Moore KN Colombo N , et al. Maintenance olaparib for patients with newly diagnosed advanced ovarian cancer and a BRCA mutation (SOLO1/GOG 3004): 5-year follow-up of a randomised, double-blind, placebo-controlled, phase 3 trial. Lancet Oncol 2021; 22: 1721–1731. 34715071 10.1016/S1470-2045(21)00531-3

[22] Ray-Coquard I Pautier P Pignata S , et al. Olaparib plus bevacizumab as first-line maintenance in ovarian cancer. N Engl J Med 2019; 381: 2416–2428. 31851799 10.1056/NEJMoa1911361

[23] Harter P Trillsch F Okamoto A , et al. Durvalumab with paclitaxel/carboplatin (PC) and bevacizumab (bev), followed by maintenance durvalumab, bev, and olaparib in patients (pts) with newly diagnosed advanced ovarian cancer (AOC) without a tumor BRCA1/2 mutation (non-tBRCAm): results from the randomized, placebo (pbo)-controlled phase III DUO-O trial. J Clin Oncol 2023; 41: LBA5506.

[24] Monk BJ Oaknin A O’Malley DM , et al. LBA30 ATHENA-COMBO, a phase III, randomized trial comparing rucaparib (RUCA) + nivolumab (NIVO) combination therapy vs RUCA monotherapy as maintenance treatment in patients (pts) with newly diagnosed ovarian cancer (OC). Ann Oncol 2024; 35: S1223–S1224.

[25] Sznurkowski JJ. To Bev or not to Bev during ovarian cancer maintenance therapy? Cancers (Basel) 2023; 15: 2980. 37296941 10.3390/cancers15112980 PMC10251846

[26] Pathak A Pal AK Roy S , et al. Role of angiogenesis and its biomarkers in development of targeted tumor therapies. Stem Cells Int 2024; 2024: 9077926. 38213742 10.1155/2024/9077926 PMC10783989

[27] Fong PC Yap TA Boss DS , et al. Poly(ADP)-ribose polymerase inhibition: frequent durable responses in BRCA carrier ovarian cancer correlating with platinum-free interval. J Clin Oncol 2010; 28: 2512–2519. 20406929 10.1200/JCO.2009.26.9589

[28] Coleman RL Fleming GF Brady MF , et al. Veliparib with first-line chemotherapy and as maintenance therapy in ovarian cancer. N Engl J Med 2019; 381: 2403–2415. 31562800 10.1056/NEJMoa1909707 PMC6941439

[29] Li N Zhu J Yin R , et al. Treatment with niraparib maintenance therapy in patients with newly diagnosed advanced ovarian cancer: a phase 3 randomized clinical trial. JAMA Oncol 2023; 9: 1230–1237. 37440217 10.1001/jamaoncol.2023.2283 PMC10346505

[30] Penson R Valencia RV Colombo N , et al. Final overall survival results from SOLO3: phase III trial assessing olaparib monotherapy versus non-platinum chemotherapy in heavily pretreated patients with germline BRCA1- and/or BRCA2-mutated platinum-sensitive relapsed ovarian cancer (026). Gynecol Oncol 2022; 166: S19–S20.

[31] Penson RT Valencia RV Cibula D , et al. Olaparib versus nonplatinum chemotherapy in patients with platinum-sensitive relapsed ovarian cancer and a germline BRCA1/2 mutation (SOLO3): a randomized phase III trial. J Clin Oncol 2020; 38: 1164–1174. 32073956 10.1200/JCO.19.02745 PMC7145583

[32] Matulonis U Herrstedt J Oza A , et al. Long-term safety and secondary efficacy endpoints in the ENGOT-OV16/NOVA phase III trial of niraparib in recurrent ovarian cancer. Gynecol Oncol 2021; 162: S24–S25. 10.1016/j.ygyno.2025.03.018 40139026

[33] Nitecki R Melamed A Gockley AA , et al. Incidence of myelodysplastic syndrome and acute myeloid leukemia in patients receiving poly-ADP ribose polymerase inhibitors for the treatment of solid tumors: a meta-analysis of randomized trials. Gynecol Oncol 2021; 161: 653–659. 33736856 10.1016/j.ygyno.2021.03.011 PMC8164998

[34] Arora S Balasubramaniam S Zhang H , et al. FDA approval summary: olaparib monotherapy or in combination with bevacizumab for the maintenance treatment of patients with advanced ovarian cancer. Oncologist 2021; 26: e164–e172. 10.1002/onco.13551 PMC7794199 33017510

[35] U.S. Food and Drug Administration. FDA approves niraparib for first-line maintenance of advanced ovarian cancer, https://www.fda.gov/drugs/resources-information-approved-drugs/fda-approves-niraparib-first-line-maintenance-advanced-ovarian-cancer (accessed 8 June 2025).

[36] Ushijima K. Treatment for recurrent ovarian cancer-at first relapse. J Oncol 2009; 2010: 497429. 20066162 10.1155/2010/497429 PMC2801501

[37] Banerjee S Gonzalez-Martin A Harter P , et al. First-line PARP inhibitors in ovarian cancer: summary of an ESMO Open – cancer horizons round-table discussion. ESMO Open 2020; 5: e001110. 10.1136/esmoopen-2020-001110 PMC7783599 33310779

[38] Harter P Marth C Mouret-Reynier MA , et al. Efficacy of subsequent therapies in patients with advanced ovarian cancer who relapse after first-line olaparib maintenance: results of the PAOLA-1/ENGOT-ov25 trial. Ann Oncol 2025; 36: 185–196. 39528049 10.1016/j.annonc.2024.10.828

[39] Harter P Sehouli J Vergote I , et al. Randomized trial of cytoreductive surgery for relapsed ovarian cancer. N Engl J Med 2021; 385: 2123–2131. 34874631 10.1056/NEJMoa2103294

[40] Lheureux S May T Wilson MK , et al. Phase II randomized multi-centre study of neoadjuvant olaparib in patients with platinum sensitive relapsed high grade serous ovarian cancer: the NEO trial. J Clin Oncol 2024; 42: 5506.

[41] Friedlander M Matulonis U Gourley C , et al. Long-term efficacy, tolerability and overall survival in patients with platinum-sensitive, recurrent high-grade serous ovarian cancer treated with maintenance olaparib capsules following response to chemotherapy. Br J Cancer 2018; 119: 1075–1085. 30353045 10.1038/s41416-018-0271-y PMC6219499

[42] Mirza MR Benigno B Dorum A , et al. Long-term safety in patients with recurrent ovarian cancer treated with niraparib versus placebo: results from the phase III ENGOT-OV16/NOVA trial. Gynecol Oncol 2020; 159: 442–448. 32981695 10.1016/j.ygyno.2020.09.006

[43] Haggstrom L Lee YC Scott C , et al. How long is long enough? An international survey exploring practice variations on the recommended duration of maintenance therapy with PARP inhibitors in patients with platinum sensitive recurrent ovarian cancer and long-term outcomes. Int J Gynecol Cancer 2024; 34: 1932–1939. 39438068 10.1136/ijgc-2024-005976

[44] Berek JS Matulonis UA Peen U , et al. Safety and dose modification for patients receiving niraparib. Ann Oncol 2018; 29: 1784–1792. 29767688 10.1093/annonc/mdy181

[45] Drew Y Ledermann J Hall G , et al. Phase 2 multicentre trial investigating intermittent and continuous dosing schedules of the poly(ADP-ribose) polymerase inhibitor rucaparib in germline BRCA mutation carriers with advanced ovarian and breast cancer. Br J Cancer 2016; 114: e21. 10.1038/bjc.2016.133 PMC4984462 27228289

[46] Sandhu SK Schelman WR Wilding G , et al. The poly(ADP-ribose) polymerase inhibitor niraparib (MK4827) in BRCA mutation carriers and patients with sporadic cancer: a phase 1 dose-escalation trial. Lancet Oncol 2013; 14: 882–892. 23810788 10.1016/S1470-2045(13)70240-7

[47] Liby K To C. Intermittent treatment with the PARP inhibitor olaparib delays tumor development in BRCA1-deficient mice [abstract]. In: Proceedings of the AACR special conference: cancer susceptibility and cancer susceptibility syndromes, 29 January–1 February 2014, vol. 74. San Diego, CA/Philadelphia (PA): AACR.

[48] Strobl MAR Martin AL West J , et al. To modulate or to skip: de-escalating PARP inhibitor maintenance therapy in ovarian cancer using adaptive therapy. Cell Syst 2024; 15: 510–525.e6. 10.1016/j.cels.2024.04.003 PMC11190943 38772367

[49] Mukhopadhyay A Ghosh T Bhattacharjee D , et al. EP290/#820 Intermittent PARP inhibitor regimen in ovarian cancer (IPIROC): origin and feasibility of implementing a proof-of-concept exploratory study. Int J Gynecol Cancer 2023; 33: A197–A198.

[50] Cecchini M Walther Z Wei W , et al. NCI 7977: a phase I dose-escalation study of intermittent oral ABT-888 (veliparib) plus intravenous irinotecan administered in patients with advanced solid tumors. Cancer Res Commun 2023; 3: 1113–1117. 37377610 10.1158/2767-9764.CRC-22-0485 PMC10292219

[51] van der Noll R Jager A Ang JE , et al. Phase I study of intermittent olaparib capsule or tablet dosing in combination with carboplatin and paclitaxel (part 2). Invest New Drugs 2020; 38: 1096–1107. 31637669 10.1007/s10637-019-00857-6

[52] Caruso G Coleman RL Aletti G , et al. Systemic therapy de-escalation in advanced ovarian cancer: a new era on the horizon? Int J Gynecol Cancer 2023; 33: 1448–1457. 37597852 10.1136/ijgc-2023-004740

[53] Gao Q-l Liu D Li H , et al. Efficacy, safety, and biomarkers of neoadjuvant niraparib monotherapy in homologous recombination deficiency (HRD) advanced ovarian cancer: a prospective, multicenter, phase II, single-arm NANT study (Meeting Abstract: 2024 ASCO Annual Meeting). J Clin Oncol 2024; 42: Abstr 5549.

[54] Westin S Michael V Fellman B , et al. Now: neoadjuvant olaparib window trial in patients with newly diagnosed BRCA mutant ovarian cancer (LBA 1). Gynecol Oncol 2023; 176: S2–S3.

[55] Harano K Nakao T Nishio S , et al. 534P A pilot study of neoadjuvant olaparib for patients with HRD-positive advanced ovarian cancer. Ann Oncol 2022; 33: S792. 10.1158/1078-0432.CCR-25-4887 42531373

[56] Harano K Nakao T Nishio S , et al. Neoadjuvant combination treatment of olaparib and pembrolizumab for patients with HRD-positive advanced ovarian cancer. J Clin Oncol 2024; 42: 5545–5545. 10.1158/1078-0432.CCR-25-4887 42531373

[57] Salutari V Marchetti C Ghizzoni V , et al. #881 The NUVOLA TRIAL: neoadjuvant chemotherapy in unresectable ovarian cancer with olaparib and weekly carboplatin plus paclitaxel. A phase II open-label multi-centre study. Int J Gynecol Cancer 2023; 33: A430–A431. 10.1136/ijgc-2021-002727 34131041

[58] Li H Liu ZY Wu N , et al. PARP inhibitor resistance: the underlying mechanisms and clinical implications. Mol Cancer 2020; 19: 107. 32563252 10.1186/s12943-020-01227-0 PMC7305609

[59] Dias MP Moser SC Ganesan S , et al. Understanding and overcoming resistance to PARP inhibitors in cancer therapy. Nat Rev Clin Oncol 2021; 18: 773–791. 34285417 10.1038/s41571-021-00532-x

[60] Mweempwa A Wilson MK. Mechanisms of resistance to PARP inhibitors – an evolving challenge in oncology. Cancer Drug Resist 2019; 2: 608–617. 35582591 10.20517/cdr.2019.50 PMC8992504

[61] Giudice E Gentile M Salutari V , et al. PARP inhibitors resistance: mechanisms and perspectives. Cancers (Basel) 2022; 14: 1420. 35326571 10.3390/cancers14061420 PMC8945953

[62] Martins MLF Loos NHC Mucuk S , et al. P-glycoprotein (ABCB1/MDR1) controls brain penetration and intestinal disposition of the PARP1/2 inhibitor niraparib. Mol Pharm 2021; 18: 4371–4384. 34730366 10.1021/acs.molpharmaceut.1c00553

[63] Wang N Yang Y Jin D , et al. PARP inhibitor resistance in breast and gynecological cancer: resistance mechanisms and combination therapy strategies. Front Pharmacol 2022; 13: 967633. 36091750 10.3389/fphar.2022.967633 PMC9455597

[64] Romeo M Gil-Martin M Gaba L , et al. Multicenter real-world data of subsequent chemotherapy after progression to PARP inhibitors in a maintenance relapse setting. Cancers (Basel) 2022; 14: 4414. 36139574 10.3390/cancers14184414 PMC9497128

[65] Cecere SC Giannone G Salutari V , et al. Olaparib as maintenance therapy in patients with BRCA 1–2 mutated recurrent platinum sensitive ovarian cancer: real world data and post progression outcome. Gynecol Oncol 2020; 156: 38–44. 31699415 10.1016/j.ygyno.2019.10.023

[66] Vacheresse G Sabri E Domingo S , et al. Response to subsequent platinum-based chemotherapy post PARP inhibitor in recurrent epithelial ovarian cancer. J Clin Oncol 2023; 41: Abstr 5578.

[67] Oza AM Lisyanskaya AS Fedenko AA , et al. Overall survival results from ARIEL4: a phase III study assessing rucaparib vs chemotherapy in patients with advanced, relapsed ovarian carcinoma and a deleterious BRCA1/2 mutation. Ann Oncol 2022; 33: S780.

[68] GOV.UK. Drug safety update rucaparib (rubraca): withdrawal of third-line treatment indication, https://www.gov.uk/drug-safety-update/rucaparib-rubracav-withdrawal-of-third-line-treatment-indication (2022, accessed 8 June 2025).

[69] EMA recommends restricting use of cancer medicine Rubraca, https://www.ema.europa.eu/en/news/ema-recommends-restricting-use-cancer-medicine-rubraca (2022, accessed 8 June 2025).

[70] Kristeleit RS Moore KN. Life after SOLO-2: is olaparib really inducing platinum resistance in BRCA-mutated (BRCAm), PARP inhibitor (PARPi)-resistant, recurrent ovarian cancer? Ann Oncol 2022; 33: 989–991. 35964823 10.1016/j.annonc.2022.08.003

[71] Pujade-Lauraine E Selle F Scambia G , et al. Maintenance olaparib rechallenge in patients with platinum-sensitive relapsed ovarian cancer previously treated with a PARP inhibitor (OReO/ENGOT-ov38): a phase IIIb trial. Ann Oncol 2023; 34(12): 1152–1164. 37797734 10.1016/j.annonc.2023.09.3110

[72] Marth C Mouret-Reynier M Lorusso D , et al. #528 Efficacy of subsequent chemotherapy followed by PARP inhibitor maintenance in patients with advanced ovarian cancer in the phase III PAOLA-1/ENGOT-Ov25 trial. Int J Gynecol Cancer 2023; 33: A21.

[73] Moore KN Secord AA Geller MA , et al. Niraparib monotherapy for late-line treatment of ovarian cancer (QUADRA): a multicentre, open-label, single-arm, phase 2 trial. Lancet Oncol 2019; 20: 636–648. 30948273 10.1016/S1470-2045(19)30029-4

[74] McMullen M Karakasis K Loembe B , et al. DUETTE: a phase II randomized, multicenter study to investigate the efficacy and tolerability of a second maintenance treatment in patients with platinum-sensitive relapsed epithelial ovarian cancer, who have previously received poly(ADP-ribose) polymerase (PARP) inhibitor maintenance treatment. Int J Gynecol Cancer 2020; 30: 1824–1828. 32878963 10.1136/ijgc-2020-001694 PMC7656147

[75] Morgan RD Clamp AR White DJ , et al. Multi-maintenance olaparib therapy in relapsed, germline BRCA1/2-mutant high-grade serous ovarian cancer (MOLTO): a phase II trial. Clin Cancer Res 2023; 29: 2602–2611. 36799931 10.1158/1078-0432.CCR-22-3282

[76] Moubarak M Harter P Ataseven B , et al. Re-treatment with PARPi in patients with recurrent epithelial ovarian cancer: a single institutional experience. Gynecol Oncol Rep 2022; 40: 100939. 35169607 10.1016/j.gore.2022.100939 PMC8829558

[77] Essel KG Behbakht K Lai T , et al. PARPi after PARPi in epithelial ovarian cancer. Gynecol Oncol Rep 2021; 35: 100699. 33537389 10.1016/j.gore.2021.100699 PMC7840844

[78] Yubero-Esteban A Reche-Molina P Coloma CS , et al. Clinical experience with rucaparib after prior PARPi treatment: a subanalysis from the rucaparib access program in Spain by GEICO. J Clin Oncol 2022; 40: Asbtr e17598.

[79] Lheureux S Oaknin A Garg S , et al. EVOLVE: a multicenter open-label single-arm clinical and translational phase II trial of cediranib plus olaparib for ovarian cancer after PARP inhibition progression. Clin Cancer Res 2020; 26: 4206–4215. 32444417 10.1158/1078-0432.CCR-19-4121

[80] Cho H Park J Kim BG , et al. PR054/#1517 A single-arm, phase II study of niraparib and bevacizumab maintenance in patients with platinum-sensitive, recurrent ovarian cancer previously treated with a PARP inhibitor (KGOG 3056/NIRVANA-R). Int J Gynecol Cancer 2023; 33: A61–A62. 10.3802/jgo.2022.33.e12 PMC8899880 34910393

[81] Wethington SL Shah PD Martin L , et al. Combination ATR (ceralasertib) and PARP (olaparib) inhibitor (CAPRI) trial in acquired PARP inhibitor-resistant homologous recombination-deficient ovarian cancer. Clin Cancer Res 2023; 29: 2800–2807. 37097611 10.1158/1078-0432.CCR-22-2444 PMC11934101

[82] Lievens Y Guckenberger M Gomez D , et al. Defining oligometastatic disease from a radiation oncology perspective: an ESTRO-ASTRO consensus document. Radiother Oncol 2020; 148: 157–166. 32388150 10.1016/j.radonc.2020.04.003

[83] Chen T Xu J Xia B , et al. Secondary cytoreduction surgery for recurrent epithelial ovarian cancer patients after PARPi maintenance: a multicenter, randomized, controlled clinical trial. Int J Gynecol Cancer 2024; 34(2): 328–331. 38159938 10.1136/ijgc-2023-004978

[84] Palluzzi E Marchetti C Cappuccio S , et al. Management of oligometastatic ovarian cancer recurrence during PARP inhibitor maintenance. Int J Gynecol Cancer 2022; 32(9): 1164–1170. 35868655 10.1136/ijgc-2022-003543

[85] Gauduchon T Kfoury M Lorusso D , et al. PARP inhibitors (PARPi) prolongation after local therapy for oligo-metastatic progression in relapsed ovarian cancer patients. Gynecol Oncol 2023; 173: 98–105. 37105063 10.1016/j.ygyno.2023.04.002

[86] Sun Z Li L Zhai B , et al. Rational design of PARP1/c-Met dual inhibitors for overcoming PARP1 inhibitor resistance induced by c-Met overexpression. J Med Chem 2024; 67: 4916–4935. 38477575 10.1021/acs.jmedchem.4c00077

[87] Kim H Xu H George E , et al. Combining PARP with ATR inhibition overcomes PARP inhibitor and platinum resistance in ovarian cancer models. Nat Commun 2020; 11: 3726. 32709856 10.1038/s41467-020-17127-2 PMC7381609

[88] Shah PD Wethington SL Pagan C , et al. Combination ATR and PARP inhibitor (CAPRI): a phase 2 study of ceralasertib plus olaparib in patients with recurrent, platinum-resistant epithelial ovarian cancer. Gynecol Oncol 2021; 163: 246–253. 34620496 10.1016/j.ygyno.2021.08.024 PMC9614917

[89] Mak J Ma H Poon R , et al. Synergism between ATM and PARP1 inhibition involves DNA damage and abrogating the G2 DNA damage checkpoint. Mol Cancer Ther 2020; 19: 123–134. 31597711 10.1158/1535-7163.MCT-19-0474

[90] Biegala L Gajek A Szymczak-Pajor I , et al. Targeted inhibition of the ATR/CHK1 pathway overcomes resistance to olaparib and dysregulates DNA damage response protein expression in BRCA2 (MUT) ovarian cancer cells. Sci Rep 2023; 13: 22659. 38114660 10.1038/s41598-023-50151-y PMC10730696

[91] Westin S Coleman R Fellman B , et al. EFFORT: EFFicacy Of adavosertib in parp ResisTance: a randomized two-arm non-comparative phase II study of adavosertib with or without olaparib in women with PARP-resistant ovarian cancer. J Clin Oncol 2021; 39: Abstr 5505.

[92] Black SJ Ozdemir AY Kashkina E , et al. Molecular basis of microhomology-mediated end-joining by purified full-length Poltheta. Nat Commun 2019; 10: 4423. 31562312 10.1038/s41467-019-12272-9 PMC6764996

[93] Mateos-Gomez PA Gong F Nair N , et al. Mammalian polymerase theta promotes alternative NHEJ and suppresses recombination. Nature 2015; 518: 254–257. 25642960 10.1038/nature14157 PMC4718306

[94] Ceccaldi R Liu JC Amunugama R , et al. Homologous-recombination-deficient tumours are dependent on Poltheta-mediated repair. Nature 2015; 518: 258–262. 25642963 10.1038/nature14184 PMC4415602

[95] Zhou J Gelot C Pantelidou C , et al. A first-in-class polymerase theta inhibitor selectively targets homologous-recombination-deficient tumors. Nat Cancer 2021; 2: 598–610. 34179826 10.1038/s43018-021-00203-x PMC8224818

[96] Zatreanu D Robinson HMR Alkhatib O , et al. Poltheta inhibitors elicit BRCA-gene synthetic lethality and target PARP inhibitor resistance. Nat Commun 2021; 12: 3636. 34140467 10.1038/s41467-021-23463-8 PMC8211653

[97] Brown JS Sundar R Lopez J. Combining DNA damaging therapeutics with immunotherapy: more haste, less speed. Br J Cancer 2018; 118: 312–324. 29123260 10.1038/bjc.2017.376 PMC5808021

[98] Peyraud F Italiano A. Combined PARP inhibition and immune checkpoint therapy in solid tumors. Cancers (Basel) 2020; 12: 1502. 32526888 10.3390/cancers12061502 PMC7352466

[99] Li T Wang X Qin S , et al. Targeting PARP for the optimal immunotherapy efficiency in gynecologic malignancies. Biomed Pharmacother 2023; 162: 114712. 37075667 10.1016/j.biopha.2023.114712

[100] Shen J Zhao W Ju Z , et al. PARPi triggers the STING-dependent immune response and enhances the therapeutic efficacy of immune checkpoint blockade independent of BRCAness. Cancer Res 2019; 79: 311–319. 30482774 10.1158/0008-5472.CAN-18-1003 PMC6588002

[101] Pantelidou C Sonzogni O De Oliveria Taveira M , et al. PARP inhibitor efficacy depends on CD8(+) T-cell recruitment via intratumoral STING pathway activation in BRCA-deficient models of triple-negative breast cancer. Cancer Discov 2019; 9: 722–737. 31015319 10.1158/2159-8290.CD-18-1218 PMC6548644

[102] Jiao S Xia W Yamaguchi H , et al. PARP inhibitor upregulates PD-L1 expression and enhances cancer-associated immunosuppression. Clin Cancer Res 2017; 23: 3711–3720. 28167507 10.1158/1078-0432.CCR-16-3215 PMC5511572

[103] Friedlander M Mileshkin L Lombard J , et al. Pamiparib in combination with tislelizumab in patients with advanced solid tumours: results from the dose-expansion stage of a multicentre, open-label, phase I trial. Br J Cancer 2023; 129: 797–810. 37474720 10.1038/s41416-023-02349-0 PMC10449784

[104] Lee JM Cimino-Mathews A Peer CJ , et al. Safety and clinical activity of the programmed death-ligand 1 inhibitor durvalumab in combination with poly (ADP-ribose) polymerase inhibitor olaparib or vascular endothelial growth factor receptor 1–3 inhibitor cediranib in women’s cancers: a dose-escalation, phase I study. J Clin Oncol 2017; 35: 2193–2202. 28471727 10.1200/JCO.2016.72.1340 PMC5493052

[105] Drew Y Kim JW Penson RT , et al. Olaparib plus durvalumab, with or without bevacizumab, as treatment in PARP inhibitor-naive platinum-sensitive relapsed ovarian cancer: a phase II multi-cohort study. Clin Cancer Res 2024; 30: 50–62. 37939124 10.1158/1078-0432.CCR-23-2249 PMC10767301

[106] Konstantinopoulos PA Waggoner S Vidal GA , et al. Single-arm phases 1 and 2 trial of niraparib in combination with pembrolizumab in patients with recurrent platinum-resistant ovarian carcinoma. JAMA Oncol 2019; 5: 1141–1149. 31194228 10.1001/jamaoncol.2019.1048 PMC6567832

[107] Ame JC Spenlehauer C de Murcia G. The PARP superfamily. Bioessays 2004; 26: 882–893. 15273990 10.1002/bies.20085

[108] Demeny MA Virag L. The PARP enzyme family and the hallmarks of cancer Part 1. Cell intrinsic hallmarks. Cancers (Basel) 2021; 13: 2042. 33922595 10.3390/cancers13092042 PMC8122967

[109] Zheng J Li Z Min W. Current status and future promise of next-generation poly (ADP-ribose) polymerase 1-selective inhibitor AZD5305. Front Pharmacol 2022; 13: 979873. 36756144 10.3389/fphar.2022.979873 PMC9899804

[110] Ronson GE Piberger AL Higgs MR , et al. PARP1 and PARP2 stabilise replication forks at base excision repair intermediates through Fbh1-dependent Rad51 regulation. Nat Commun 2018; 9: 746. 29467415 10.1038/s41467-018-03159-2 PMC5821833

[111] Murai J Huang SY Das BB , et al. Trapping of PARP1 and PARP2 by clinical PARP inhibitors. Cancer Res 2012; 72: 5588–5599. 23118055 10.1158/0008-5472.CAN-12-2753 PMC3528345

[112] Johannes JW Balazs A Barratt D , et al. Discovery of 5-4-[(7-ethyl-6-oxo-5,6-dihydro-1,5-naphthyridin-3-yl)methyl]piperazin-1-yl-N-methylpyridine-2-carboxamide (AZD5305): a PARP1-DNA trapper with high selectivity for PARP1 over PARP2 and other PARPs. J Med Chem 2021; 64: 14498–14512. 34570508 10.1021/acs.jmedchem.1c01012

[113] Yap TA Im SA Schram AM , et al. Abstract CT007: PETRA: first in class, first in human trial of the next generation PARP1-selective inhibitor AZD5305 in patients (pts) with BRCA1/2, PALB2 or RAD51C/D mutations. Cancer Res 2022; 82: CT007.

[114] Rustin GJ Vergote I Eisenhauer E , et al. Definitions for response and progression in ovarian cancer clinical trials incorporating RECIST 1.1 and CA 125 agreed by the Gynecological Cancer Intergroup (GCIG). Int J Gynecol Cancer 2011; 21: 419–423. 21270624 10.1097/IGC.0b013e3182070f17

[115] Zebic DS Tjokrowidjaja A Francis KE , et al. Discordance between GCIG CA-125 progression and RECIST progression in the CALYPSO trial of patients with platinum-sensitive recurrent ovarian cancer. Br J Cancer 2024; 130: 425–433. 38097739 10.1038/s41416-023-02528-z PMC10844635

[116] Tjokrowidjaja A Lee CK Friedlander M , et al. Concordance between CA-125 and RECIST progression in patients with germline BRCA-mutated platinum-sensitive relapsed ovarian cancer treated in the SOLO2 trial with olaparib as maintenance therapy after response to chemotherapy. Eur J Cancer 2020; 139: 59–67. 32977221 10.1016/j.ejca.2020.08.021

[117] Tjokrowidjaja A Friedlander ML Ledermann JA , et al. Poor concordance between cancer antigen-125 and RECIST assessment for progression in patients with platinum-sensitive relapsed ovarian cancer on maintenance therapy with a poly(ADP-ribose) polymerase inhibitor. J Clin Oncol 2024; 42: 1301–1310. 38215359 10.1200/JCO.23.01182

[118] Alaggio R Amador C Anagnostopoulos I , et al. Correction: ‘The 5th edition of the world health organization classification of haematolymphoid tumours: lymphoid neoplasms.’ Leukemia. 2022 Jul;36(7):1720–1748. Leukemia 2023; 37: 1944–1951. 35732829 10.1038/s41375-022-01620-2 PMC9214472

[119] Morice PM Leary A Dolladille C , et al. Myelodysplastic syndrome and acute myeloid leukaemia in patients treated with PARP inhibitors: a safety meta-analysis of randomised controlled trials and a retrospective study of the WHO pharmacovigilance database. Lancet Haematol 2021; 8: e122–e134. 10.1016/S2352-3026(20)30360-4 33347814

[120] Caruso G Gigli F Parma G , et al. Myeloid neoplasms post PARP inhibitors for ovarian cancer. Int J Gynecol Cancer 2023; 33: 598–606. 36707087 10.1136/ijgc-2022-004190

[121] Navitski A Al-Rawi DH Liu Y , et al. Baseline risk of hematologic malignancy at initiation of frontline PARP inhibitor maintenance for BRCA1/2-associated ovarian cancer. Gynecol Oncol Rep 2021; 38: 100873. 34926756 10.1016/j.gore.2021.100873 PMC8651772

[122] Morice PM Ray-Coquard I Moore KN , et al. PARP inhibitors and newly second primary malignancies in cancer patients: a systematic review and safety meta-analysis of placebo randomized controlled trials. Ann Oncol 2021; 32: 1048–1050. 34107345 10.1016/j.annonc.2021.04.023

[123] Vergote I Gonzalez-Martin A Lorusso D , et al. Clinical research in ovarian cancer: consensus recommendations from the Gynecologic Cancer InterGroup. Lancet Oncol 2022; 23: e374–e384. 10.1016/S1470-2045(22)00139-5 PMC9465953 35901833

[124] Ledermann J Harter P Gourley C , et al. Olaparib maintenance therapy in platinum-sensitive relapsed ovarian cancer. N Engl J Med 2012; 366: 1382–1392. 22452356 10.1056/NEJMoa1105535

[125] Nicum S Blagden SP. PARPs: all for one and one for all? Enhancing diversity in clinical trials. Clin Cancer Res 2022; 28: 2201–2203. 35357469 10.1158/1078-0432.CCR-22-0442

[126] Wu XH Zhu JQ Yin RT , et al. Niraparib maintenance therapy in patients with platinum-sensitive recurrent ovarian cancer using an individualized starting dose (NORA): a randomized, double-blind, placebo-controlled phase III trial. Ann Oncol 2021; 32: 512–521. 33453391 10.1016/j.annonc.2020.12.018

[127] Wu X Wu L Kong B , et al. The first nationwide multicenter prevalence study of germline BRCA1 and BRCA2 mutations in Chinese ovarian cancer patients. Int J Gynecol Cancer 2017; 27: 1650–1657. 28692638 10.1097/IGC.0000000000001065

[128] Shao D Cheng S Guo F , et al. Prevalence of hereditary breast and ovarian cancer (HBOC) predisposition gene mutations among 882 HBOC high-risk Chinese individuals. Cancer Sci 2020; 111: 647–657. 31742824 10.1111/cas.14242 PMC7004523

[129] Wu X Liu J Wang J , et al. Senaparib as first-line maintenance therapy in advanced ovarian cancer: a randomized phase 3 trial. Nat Med 2024; 30: 1612–1621. 38750351 10.1038/s41591-024-03003-9

[130] Wagar MK Mojdehbakhsh RP Godecker A , et al. Racial and ethnic enrollment disparities in clinical trials of poly(ADP-ribose) polymerase inhibitors for gynecologic cancers. Gynecol Oncol 2022; 165: 49–52. 35144798 10.1016/j.ygyno.2022.01.032

[131] Yu Y Durairaj C Shi H , et al. Population pharmacokinetics of talazoparib in patients with advanced cancer. J Clin Pharmacol 2020; 60: 218–228. 31489639 10.1002/jcph.1520

[132] Committee for Medicinal Products for Human Use (CHMP) European Medicines Agency (EMA). Public assessment report rucaparib, https://www.ema.europa.eu/en/documents/variation-report/rubraca-h-c-4272-ii-0036-assessment-report-variation_en.pdf (accessed 8 June 2025).

[133] Zhang J Zheng H Gao Y , et al. Phase I pharmacokinetic study of niraparib in Chinese patients with epithelial ovarian cancer. Oncologist 2020; 25: 19.e10. 10.1634/theoncologist.2019-0565 PMC6964144 31439812

[134] Kurian AW Ward KC Howlader N , et al. Genetic testing and results in a population-based cohort of breast cancer patients and ovarian cancer patients. J Clin Oncol 2019; 37: 1305–1315. 30964716 10.1200/JCO.18.01854 PMC6524988

[135] Liposits G Wulff CN Otland A , et al. Olaparib treatment in older patients with ovarian cancer: need for ‘real-world’ data beyond clinical trials. Ecancermedicalscience 2020; 14: 1104. 33082854 10.3332/ecancer.2020.1104 PMC7532029

[136] Shi S Klotz U. Age-related changes in pharmacokinetics. Curr Drug Metab 2011; 12: 601–610. 21495970 10.2174/138920011796504527

[137] Antherieu G Heiblig M Freyer G , et al. Impact of age on poly(ADP-ribose) polymerase inhibitor (PARPi)-induced lymphopenia: a scoping review of the literature and internal analysis of a retrospective database. Drugs Aging 2023; 40: 397–405. 37081248 10.1007/s40266-023-01023-7 PMC10118227

[138] Li CH Haider S Boutros PC. Age influences on the molecular presentation of tumours. Nat Commun 2022; 13: 208. 35017538 10.1038/s41467-021-27889-y PMC8752853

